# Lethal and Sublethal Effects of Essential Oils from *Eucalyptus camaldulensis* and *Heracleum persicum* Against the Adults of Callosobruchus Maculatus

**DOI:** 10.1673/031.013.15201

**Published:** 2013-12-17

**Authors:** Khadijeh Izakmehri, Moosa Saber, Ali Mehrvar, Mohammad Bagher Hassanpouraghdam, Samad Vojoudi

**Affiliations:** 1 Department of Plant Protection, College of Agriculture, University of Maragheh, Maragheh, Iran; 2 Department of Plant Protection, College of Agriculture, Azarbaijan, Shahid Madani University, Tabriz, Iran; 3 Department of Horticulture, College of Agriculture, University of Maragheh, Maragheh, Iran

**Keywords:** botanicals, cowpea weevil, fecundity, fertility, fumigant effects

## Abstract

The cowpea weevil, *Callosobruchus maculatus* F. (Coleoptera: Bruchidae), is an important pest of stored cowpea, *Vigna ungiculata* (L.) Walpers (Fabales: Fabaceae), with ample distribution in tropical and subtropical regions. Many plant essential oils have a broad-spectrum activity against pest insects, and these oils traditionally have been used in the protection of stored products. In this study, the lethal and sublethal effects of essential oils from *Eucalyptus camaldulensis* Dehnh. (Myrtales: Myrtaceae) *and Heracleum persicum* Desf. (Apiales: Apiaceae) were evaluated on the adults of *C. maculatus* at 26 ± 1° C, 70 ± 5% RH, and a photoperiod of 16:8 L:D. The LC_50_ values of *E. camaldulensis* and *H. persicum* were 56.7 and 219.4 µL/L air after 12 hr and 26.1 and 136.4 µL/L air after 24 hr of exposure, respectively. The LT_50_ values of *E. camaldulensis* and *H.persicum* were 6.3 and 10.9 hr, respectively. The results showed that low lethal concentration (LC_20_) of essential oils negatively affected the longevity, fecundity, and fertility of female adults. The sex ratio of *C. maculatus* offspring was not significantly affected by essential oils. Therefore, these essential oils can be suggested for controlling *C. maculatus* in storage systems. The introduction of essential oils into storage systems could potentially decrease seed losses.

## Introduction

The cowpea weevil, *Callosobruchus maculatus* F. (Coleoptera: Bruchidae), is an important pest of stored grains such as cowpea, lentil, chickpea, and other legumes, with ample distribution in tropical and subtropical regions, where those crops represent one of the main protein sources for humans (Keita et al. 2001; Appleby and Credland 2003; Lima et al. 2004). *C. maculatus* infests cowpea before harvest and causes quantitative and qualitative seed losses in storage facilities (Ajayi and Laie 2001; Lima et al. 2004; Erler et al. 2009). The cowpea weevil larvae feed inside the grain seeds and cause weight losses of up to 80% after six months in storage (Aboua et al. 2010). The control of this pest in storage systems mainly depends on insecticides and fumigants, such as deltamethrin, malathion, phosphine, and methyl bromide (Isman 2000; Ajayi and Laie 2001; Appleby and Credland 2003; Erler et al. 2009; Aboua et al. 2010; Manzoomi et al. 2010; Nyamador et al. 2010). However, the use of conventional insecticides and fumigant compounds has serious side effects, such as the development of insecticide resistance in the treated pest, toxic residue problems, toxicity to humans, and increasing costs of application (Aboua et al. 2010; Mollaei et al. 2011). Fumigation with synthetic insecticides has been used to control insects in commercial storage centers, but the availability and cost of these chemicals limit their common use by farmers (Ketoh et al. 2002). The use of methyl bromide was banned in many countries starting in 2004 due to its ozone depleting properties (Ketoh et al. 2002; Lee et al. 2008; Ayvaz et al. 2010). In addition, the environmental problems caused by overuse of pesticides have been a matter of concern for both scientists and the general public in recent years (Koul et al. 2008).

There is an urgent need to develop safe alternatives with the potential to replace the conventional insecticides and toxic fumigants used against stored-product insect pests (Ketoh et al. 2002; Jenkins et al. 2003; Mollaei et al. 2011). Some plant taxa, such as Myrtaceae, Lauraceae, Rutaceae, Lamiaceae, Asteraceae, Apiaceae, Cupressaceae, Poaceae, Zingiberaceae, and Piperaceae, are known to possess insecticidal properties owing to the essential oil fraction (Tripathi et al. 2009; Ayvaz et al. 2010). The biological activities of essential oils are influenced by the chemical composition and the bruchid insect developmental stage (Nyamador et al. 2010). Plant-derived materials are more readily biodegradable. Some are less toxic to mammals, may be more selective in action, and may slow the development of resistance (Rahman and Talukder 2006). Their other advantage is that they can be easily and cheaply produced by farmers and small-scale industries as crude or partially purified extracts (Keita et al. 2001; Rahman and Talukder 2006; Nyamador et al. 2010). Much research about managing *C. maculatus* with various essential oils has been conducted (Tripathi et al. 2003b; Ayvaz et al. 2010; Manzoomi et al. 2010; Zapata and Smagghe 2010). Most of the essential oil constituents are monoterpenoids and sesquiterpenoids. Essential oils from different plant species possess ovicidal, larvicidal, and repellent properties against various insect species and are regarded as environmentally compatible pesticides (Isman 2000). In addition to their lethal effect on adult and juvenile stages of insects, essential oils and their constituents also present different sublethal effects (Alzogaray et al. 2011). Botanicals used as insecticides presently constitute 1% of the world insecticide market (Ayvaz et al. 2010). It has been reported that when mixed with stored grains, leaf, bark, seed powder, or oil, extracts of plants reduce the oviposition rate, suppress adult emergence of bruchids, and reduce the seed damage rate (Keita et al. 2001; Rahman and Talukder 2006).

The main aim of this study was to evaluate the lethal and sublethal effects of essential oils from the red river gum, *Eucalyptus camaldulensis* Dehnhardt (Myrtales: Myrtaceae), and golpar, *Heracleum persicum* Destontaines (Apiales: Apiaceae), on *C. maculatus* under laboratory conditions. The sublethal effects of the essential oils, such as effects on fecundity, fertility, longevity, and sex ratio, were evaluated on *C. maculatus.*

## Materials and Methods

### Plant materials

Foliage of *E. camaldulensis* and *H. persicum* was collected during full flowering stage from Ramsar, Iran, in 2010. Plant materials were dried naturally on laboratory benches at room temperature (23–24° C) for 5 days. The dried material was stored in sealed bags until essential oil was extracted. Dried leaves and seeds were employed for the essential oil extraction of *E. camaldulensis* and *H. persicum,* respectively (Negahban et al. 2006).

### Extraction of essential oils

A sample (50 g) of air-dried powdered plant material was extracted by the hydrodistillation technique during 3 hours in an all-glass clevenger-type apparatus (Has-sanpouraghdam et al. 2009). The extracted crude essential oil was dried over anhydrous sodium sulphate and stored in a hermetically sealed glass flask with a rubber lid, covered with aluminum foil to protect the contents from photo-conversion, and kept under refrigeration at 4° C until analysis.

### Insect rearing

A culture of *C. maculatus* was obtained from the Department of Entomology, University of Tarbiat Modares, Iran, in 2011. The weevils were reared on cowpea for three generations. Fifty pairs of 1- or 2-day-old *C. maculatus* adults were then transferred and kept for 24 hr on 150 g seeds of *Vigna ungiculata* (L.) (Fabales: Fabaceae) in a plastic jar of 2000 mL volume in a growth chamber at 26 ± 1° C, 70 ± 5% RH, and a photoperiod of 16:8 L:D. The females laid eggs on these seeds, and their offspring completed their developmental stage within the cotyledons. Old seeds were replaced by new ones monthly to prevent crowding of the buchids. Under these conditions, the mean development time varied between 30 and 35 days. The adults were isolated after emergence and used either for the production of a new generation or for the rest of the experiments. Adult insects (< 24 hr old) were used for fumigant toxicity tests.

### Evaluation of the essential oils fumigant insecticidal activity

Bioassay tests were carried out to assess LC_50_ values. The concentration-setting experiments were conducted to determine the main concentrations needed for LC_50_ studies for both essential oils (Robertson et al. 2007). Essential oils were diluted with acetone. Filter paper (2 cm diameter) was soaked in pure oil at concentrations calculated to produce the fumigant. It was then put on the top of a glass vial, and a cloth mesh was used to prevent insects from direct contact with the oil. Ten adult insects (< 24 hr old) of *C. maculatus* were introduced into 33 mL glass bottles with screw lids. The main concentrations of the oils tested on *C. macnlatus* were 18.2, 24.2, 30.3, 45.5, 60.6, 75.8, and 90.9 µL/L air for *E. camaldulensis* and 84.8, 121.2, 157.6, 193.9, 230.3, 266.7, and 303 µL/L air for *H. persicum* for 12 hr, and 6.1, 12.1, 18.2, 24.2, 30.3, 45.5, and 60.6 µL/L air for *E. camaldulensis* and 60.6, 90.9, 121.2, 151.5, 181.2, 212.1, and 242.4 µL/L air for *H. persicum* for 24 hr, respectively.

The control consisted of the same experi-mental unit without the addition of any substance. Each concentration consisted of three replications, and each bioassay test was replicated three times. Dead insects and those that appeared extremely lethargic or unable to maintain equilibrium were record-ed as dead. In order to determine LC_50_ values, mortality was recorded after 12 and 24 hr. Data were analyzed using Probit analysis (SAS Institute 2002).

A bioassay was designed to determine the median effective time to cause mortality of 50% of test insects (LT_50_ value) at 60.6 and 242.4 µL/L air for *E. camaldulensis* and *H. persicum* respectively. These concentrations were used because they showed high mortality in the test, and usually the highest concentration of any bioassay test is used for calculating lethal time. Five pairs of newly emerged adults of *C. maculatus* were introduced into each 33 mL glass jar. The mortality was assessed by direct observation of the insects every 1, 3, 6, 9, 12, 15, 18, and 24 hr for up to the end point of mortality. The experiment was replicated three times. The data were analyzed using Proc Probit (SAS Institute 2002). The LC_50_ and LT_50_ values of the essential oils were considered significantly different if the fiducial limits did not overlap.

### Sublethal effects

To determine the sublethal effects of the *E. camaldulensis* and *H. persicum* oils, 50 female adults (< 24 hr old) of *C. maculatus* were exposed to an LC_20_ of each essential oil for a period of 24 hr in jars. The LC_20_ was chosen as a low lethal concentration because it was almost close to the mortality threshold (30%) recommended for the use of essential oils in integrated pest management (Desneux et al, 2007). The concentrations equivalent to LC_20_ were 14.2 and 78.8 µL/L air for *E. camaldulensis* and *H. persicum* oil, respectively. In control containers, no essential oil was used. The live insects in each bottle were counted 24 hr after initial exposure to either essential oil, transferred individually to a microtube with 1 cowpea seed, and then paired with a young male to ensure sufficient mating during their life span. The daily survival of each individual and the number of eggs laid by each female were recorded. Each individual was daily transferred into a new microtube with a new seed. The total number of eggs and the sex of the emerged beetles at adulthood were recorded as well.

The number of eggs laid, egg hatch rate, and offspring sex ratio were recorded for all individuals. The data were analyzed by analysis of variance with mean separation at a 5% level of significance by the least significant difference test.

### Determination of oviposition deterrence

The oviposition deterrence studies were performed by applying *E. camaldulensis* and *H. persicum* essential oils in specific doses of each on chickpea seeds, which were then kept with the controls. Each treatment was replicated three times. About 10 g of seeds were stored in glass jars (16 × 8 cm), and two pairs (two female with two male) of newly emerged (< 24 hr old) *C. maculatus* were introduced in each container. Untreated seeds were used as controls. After 15 days, the number of eggs laid on treated and control seeds were recorded, and the percentage of oviposition deterrence was calculated by using the following formula:


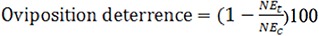


Where NE_t_ = Number of eggs laid on treated seeds, and NE_C_ = Number of eggs laid on control seeds

**Table 1.**
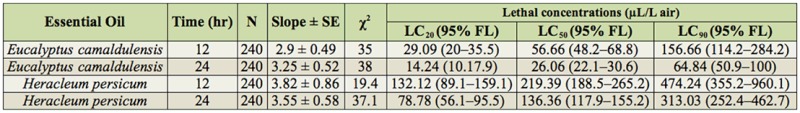
Fumigant toxicity of two essential oils, *Eucalyptus camaldulensis* and *Heracleum persicum*, on adult *Callosobruchus maculatus* after 12 and 24 hr.

**Table 2.**
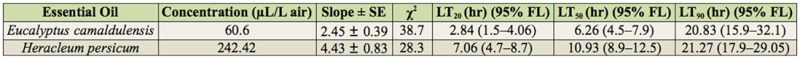
LT_50_ values of two essential oils, *Eucalyptus camaldulensis* and *Heracleum persicum*, on adult *Callosobruchus maculatus* after 24 hr.

**Table 3.**
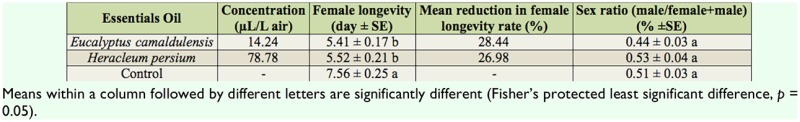
Sublethal effects of LC_20_ values of two essential oils, *Eucalyptus camaldulensis* and *Heracleum persicum*, on longevity and sex ratio of *Callosobruchus maculatus*.

**Table 4.**
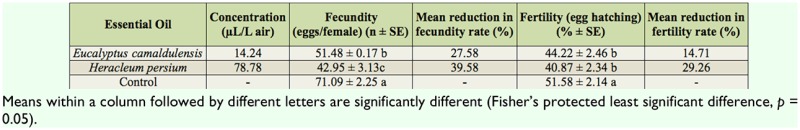
Sublethal effects of LC_20_ values of two essential oils, *Eucalyptus camaldulensis* and *Heracleum persicum*, on fecundity and fertility of *Callosobruchus maculatus*.

## Results

### Evaluation of the essential oils fumigant insecticidal activity

Experiments were conducted to determine whether the insecticidal activity of *E. camaldulensis* and *H. persicum* oils against *C. maculatus* adults was attributable to fumigant action. In all cases, a considerable difference in the mortality of insects to essential oil vapors was observed with different concentrations and times. The LC_50_ values indicated that *E. camaldulensis* oil was more effective than *H. persicum* oil at both 12 and 24 hr exposure (Table 1). The LT_50_ results of insecticides are shown in Table 2. These results showed that *E. camaldulensis* oil affected the insects faster than *H. persicum* oil.

**Figure 1.**
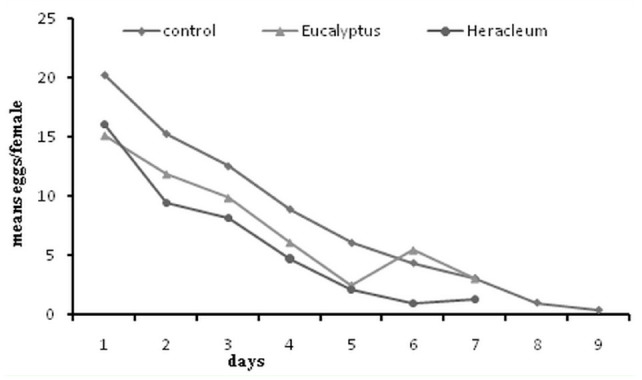
Daily female progeny production by adult *Callosobruchus maculatus* treated with an LC_20_ of essential oils from *Eucalyptus camaldulensis* and *Heracleum persicum*. High quality figures are available online.

**Figure 2.**
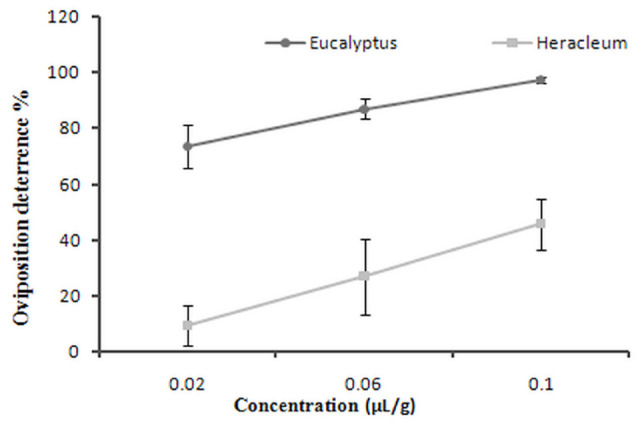
The oviposition deterrents of *Callosobruchus maculatus* after exposure to different concentrations of essential oils from *Eucalyptus camaldulensis* and *Heracleum persicum*. High quality figures are available online.

### Sublethal effects

The sublethal effects of the essential oils on longevity, fecundity, fertility, and sex ratio of *C. maculatus* females are shown in Tables 3 and 4. The longevity, fecundity, and fertility of female adults were affected significantly by the essential oils, but sex ratio was not (longevity: *F=* 32.2; df = 2*; p* = 0.0001; fecundity: *F =* 25.2; df = 2; *p =* 0.0001; fertility: *F* = 12.5; df = 2; *p =* 0.0001; sex ratio: *F* = 1.76; df = 2; *p =* 0.181). Adult longevity was reduced by 28.4% and 27% by essential oils of *E. camaldulensis* and *H. persicum* respectively compared to the control. Female fecundity was affected significantly and reduced by 27.6% and 39.6% in *E. camaldulensis* and *H. persicum* treatments, respectively. Both essential oils had a significant effect on the oviposition behavior of *C. maculatus* adults. Daily adult female progeny production is shown in Figure 1.

### Oviposition deterrence

The mean percentage of oviposition deterrence of *C. maculatus* after exposure to different concentrations of essential oils is shown in Figure 2. Concentrations of *E. camaldulensis* had significant oviposition deterrence effects, but there was not a significant difference between the concentrations. *E. camaldulensis* showed a higher oviposition deterrent property compared to *H. persicum* (F = 18.8, df = 5, *p <* 0.001) *H. persicum* showed a significant effect on adult oviposition (*F* = 18.8; df = 5; *p* = 0. 001), and the effects were increased by increasing the concentration.

## Discussion

Essential oils are phytochemicals with large insecticidal activities (Lee et al. 2008; Chu et al. 2011). Due to their high volatility under ambient temperatures, they have fumigant activity that might be important for controlling stored-product insect pests (Tripathi et al. 2002; Tripathi et al. 2003a; Ayvaz et al. 2010). In our study, the crude hydrodistilled essential oils obtained from *E. camaldulensis* and *H. persicum* showed potential insecticidal activity against adult *C. maculatus.* The fumigant activity of *E. camaldulensis* essential oil was higher than *H. persicum.* The insecticidal activity of the essential oils usually varies depending on the stage of the insect, the species, and the plant origin of the essential oil that would be attributed to the diverse chemical composition of the oils and the interactions that may occur between the individual components of the mixture (Negahban et al. 2006; Ayvaz et al. 2010; Alzogaray et al. 2011). Lee et al. (2008) evaluated the toxicity of two cassia and four cinnamon oils on adult *Si tophi his oryzae* by residual and vapor toxicity bioassays. In residual bioassay, cassia and cinnamon oils exhibited good insecticidal activity. Their results showed that the toxicity of the compounds was largely due to their action in the vapor phase. The fumigant toxic effects of different essential oils were assessed on the adults of *C. maculatus* as well (Keita et al. 2001; Raja and William 2008; Aboua et al. 2010). Srivastava et al. (1988) reported that eucalyptus oil effectively prevented the oviposition of insects. Tripathi et al. (2002) showed the contact and fumigant controlling effect of the leaf essential oil of *Curcuma longa* (Var. Ch-66) on three stored-product beetles, *Rhyzopertha dominica* (lesser grain borer), *Sitophilus oryzae* (rice weevil), and *Tribolium castaneum* (red flour beetle). They showed that *C. longa* leaf oil reduced the growth rate and food consumption in adults of *R. dominica* and *S. oryzae*, and larvae and adults of *T. castaneum.*

In our study, sub-lethal doses of *E. camaldulensis* and *H. persicum* essential oils reduced the longevity and fecundity of *C. maculatus.* The longevity and fecundity of adult females were affected significantly by the essential oils. The effect of essential oils on fecundity was more pronounced, and hence there was a significant difference in fertility between the control and the treatments. Female fertility was reduced by 14.7% and 21.2% in *E. camaldulensis* and *H. persicum* treatments, respectively. Both oils had a significant effect on the oviposition behavior of *C. maculatus* adults. Ketoh et al. (2002) showed the susceptibility of the bruchid *C. maculatus* and its parasitoid *Dinarmus basalis* to essential oils from *Cymbopogon nardus, Cymbopogon schoenanthus*, and *Ocimum basilicum.* They also noted that after exposure of *C. maculatus* females to the concentration of 0.5 µ/L of the essential oils, the fecundity and adult longevity were significantly reduced compared to the control. Keita et al. (2001) showed the essential oils from *Ocimum basilicum* and *O. gratissimum* affected the egg hatch rate and emergence of adult *C. maculatus.*

In the present study, oviposition was significantly reduced by *E. camaldulensis* oil, and was also significantly reduced by *H. persicum* oil. Oviposition deterrence of different concentration levels of oil of *E. camaldulensis* was more than 50% on the pest. Singh (2011) studied the effects of plant products on oviposition deterrence of *C. maculatus.* The different doses of the essential oils showed oviposition deterrence ranged from 44.2 to 47.4%. The mean oviposition deterrent property ranged from 45.01 to 48.40%, depending on whether it was plant extracts or powders.

These results indicated that *E. camaldulensis* and *H. persicum* essential oils can be considered as useful alternatives for insect control chemicals. Most essential oils have environmentally friendly properties and can be considered as reduced-risk pesticides. However, for the practical use of these plant essential oils, further studies on their safety to human health are necessary. In addition, research on the different formulations may lead to improvements in the effectiveness of repellency and potency in fumigant activity against insects of economic importance. Overall, the present study indicated that both the essential oils tested had high potential for controlling *C. maculatus.*
